# The role of nicotinic receptors in SARS-CoV-2 receptor ACE2 expression in intestinal epithelia

**DOI:** 10.1186/s42234-020-00057-1

**Published:** 2020-10-28

**Authors:** Anne S. ten Hove, David J. Brinkman, Andrew Y. F. Li Yim, Caroline Verseijden, Theo B. M. Hakvoort, Iris Admiraal, Olaf Welting, Patricia H. P. van Hamersveld, Valérie Sinniger, Bruno Bonaz, Misha D. Luyer, Wouter J. de Jonge

**Affiliations:** 1 Tytgat Institute for Liver and Intestinal Research, Amsterdam University Medical Centers, University of Amsterdam, Gastroenterology and Hepatology, Amsterdam Gastroenterology Endocrinology Metabolism, Amsterdam, 1105 BK the Netherlands; 2 Department of Surgery, Catharina Hospital, 5623 EJ Eindhoven, the Netherlands; 3 Department of Clinical Genetics, Genome Diagnostics Laboratory, Amsterdam Reproduction and Development, Amsterdam University Medical Centers, University of Amsterdam, Amsterdam, 1105 AZ the Netherlands; 4 Grenoble Institute of Neurosciences, Division of Hepato-Gastroenterology, University Grenoble Alpes, Inserm U1216, 38000 Grenoble, France; 5 Department of General, Visceral-, Thoracic and Vascular Surgery, University Hospital Bonn, 53127 Bonn, Germany

**Keywords:** Vagus nerve stimulation, nAChR, COVID-19, SARS-CoV-2, ACE2, TMPRSS2

## Abstract

**Background:**

Recent evidence demonstrated that severe acute respiratory syndrome coronavirus 2 (SARS-CoV-2) propagates in intestinal epithelial cells expressing Angiotensin-Converting Enzyme 2 (ACE2), implying that these cells represent an important entry site for the viral infection. Nicotinic receptors (nAChRs) have been put forward as potential regulators of inflammation and of ACE2 expression. As vagus nerve stimulation (VNS) activates nAChRs, we aimed to investigate whether VNS can be instrumental in affecting intestinal epithelial *ACE2* expression.

**Methods:**

By using publicly available datasets we qualified epithelial *ACE2* expression in human intestine, and assessed gene co-expression of *ACE2* and SARS-CoV-2 priming *Transmembrane Serine Protease 2 (TMPRSS2)* with nAChRs in intestinal epithelial cells. Next, we investigated mouse and human *ACE2* expression in intestinal tissues after chronic VNS via implanted devices.

**Results:**

We show co-expression of *ACE2* and *TMPRSS2* with nAChRs and α7 nAChR in particular in intestinal stem cells, goblet cells, and enterocytes. However, VNS did not affect *ACE2* expression in murine or human intestinal tissue, albeit in colitis setting.

**Conclusions:**

*ACE2* and *TMPRSS2* are specifically expressed in epithelial cells of human intestine, and both are co-expressed with nAChRs. However, no evidence for regulation of *ACE2* expression through VNS could be found. Hence, a therapeutic value of VNS with respect to SARS-CoV-2 infection risk through ACE2 receptor modulation in intestinal epithelia could not be established.

## Background

Coronavirus disease 2019 (COVID-19), caused by severe acute respiratory syndrome coronavirus 2 (SARS-CoV-2), is characterized by a vast release of cytokines. Aggravated by following sepsis, this was established to be the cause of death in 28% of the infected patients (Zhang et al. 2020). At the time of writing this manuscript, no cure nor vaccine exists yet and hence, many studies investigating treatment options have been initiated. Amongst these, vagus nerve stimulation (VNS) has been put forward as potential therapy because of its ability to induce an anti-inflammatory effect through dampening systemic inflammatory responses. This ‘cholinergic anti-inflammatory pathway’ of the vagus nerve has been acknowledged for many years, in particular at the level of sepsis-related cytokines Tumor Necrosis Factor (TNF), Interleukin (IL)-1, and High Mobility Group Box 1 (HMBG1) (Tracey 2002). Therefore, since the COVID-19 outbreak late 2019, several trials (e.g. ClinicalTrials.gov; NCT04341415, NCT04368156, NCT04382391) have been originated to study the efficacy of electrical VNS as treatment for this hyper-inflammatory disease (Staats et al. 2020; Fudim et al. 2020). However, clear evidence supporting the value of VNS in treating COVID-19 is lacking.

Thus far, it has been demonstrated that SARS-CoV-2 uses Angiotensin-Converting Enzyme 2 (ACE2) as the key receptor for entry and Transmembrane Serine Protease 2 (TMPRSS2) for viral spike protein priming (Hoffmann et al. 2020). In addition, increasing evidence suggests that nicotinic receptors (nAChRs) have a pivotal role in the pathogenesis of COVID-19. For instance, an unusually low prevalence of cigarette smoking (characterized by prolonged nAChR activation) was clinically observed in COVID-19 patients (Creamer et al. 2019; Petrilli et al. 2020). This was confirmed by various systematic reviews showing the protective effect of current or former smoking habit against COVID-19 hospitalization (Farsalinos et al. 2020; Team CC-R 2020).

Although ACE2 is abundantly present in lung alveolar epithelial and ciliated cells, the highest ACE2 expression in the human body was found in intestinal enterocytes (Hamming et al. 2004; Qi et al. 2020). Intriguingly, nAChRs are equally expressed by enterocytes in the intestinal epithelium (Richardson et al. 2003; Summers et al. 2003). The gastrointestinal tract was therefore introduced as target organ for SARS-CoV-2 infection with up to 34% of COVID-19 patients reporting digestive symptoms like diarrhea, nausea, and abdominal discomfort (Yang and Tu 2020). The virus can propagate through ACE2 expressing enterocytes, and viral RNA can be found in rectal swabs, even after negative nasopharyngeal testing (Lamers et al. 2020; Amirian 2020; Xiao et al. 2020).

The aim of this study is to delineate ACE2 expression in intestinal epithelial cells, and to evaluate the expression of nAChRs in relation to ACE2 in intestinal epithelium. Further, we address the potency of VNS in the reduction of SARS-CoV-2 transmission. To this end, we made use of intestinal tissues of mice and humans that underwent chronic electrical non-invasive VNS administered via implanted devices (Brinkman et al., manuscript in preparation) (Bonaz et al. 2016; Sinniger et al. 2020).

## Methods

### Receptor expression assessment

Publicly available single-cell RNA-sequencing data from ileal biopsies obtained from Crohn’s disease patients (GSE134809) was downloaded from the Sequence Read Archive (SRA) whereupon they were aligned against GRCh38 using Cellranger (v3.1.0) and imported into the R statistical environment (v3.6.3) (Martin et al. 2019; R Core Team 2016). Seurat (v3.1.5) was used to import, integrate, and cluster the data (Butler et al. 2018; Stuart et al. 2019). Data visualization was done in ggplot2 (v3.3.1) (Wickham 2016). Louvain clustering analysis identified 22 cell clusters, with clusters 8, 11, 13, 14, and 18 likely representing the epithelial cells based on their expression of *CDH1* and *VIL1*. The epithelial clusters were first filtered for dead cells, identified on the basis of a low gene count and high (> 25%) percentage of mitochondrial DNA, whereupon the cell cycle effects were regressed out using the cell cycle genes (Nestorowa et al. 2016). The epithelial cells were subjected to another round of clustering analysis to identify epithelial subsets (Luecken and Theis 2019). Specifically, we identified stem cells (*LGR5*, *ASCL2*, *OLFM4*, *GKN3*, *SLC12A2*, *AXIN2*), goblet cells (*MUC2*, *TFF3*, *CLCA3*, *AGR2*), enterocytes (*FABP1*, *ALPI*, *APOA1*, *APOA4*), enteroendocrine cells (*CHGA*, *CHGB*, *TAC1*, *TPH1*, *NEUROG3*), and tuft cells (*DCLK1*, *TRPM5*, *GFI1B*, *IL25*) (Haber et al. 2017; Grun et al. 2015). Subsequently, co-expression of *ACE2* and *TMPRSS2* with nAChRs was assessed.

### VNS in humans

**Fig. 1:**
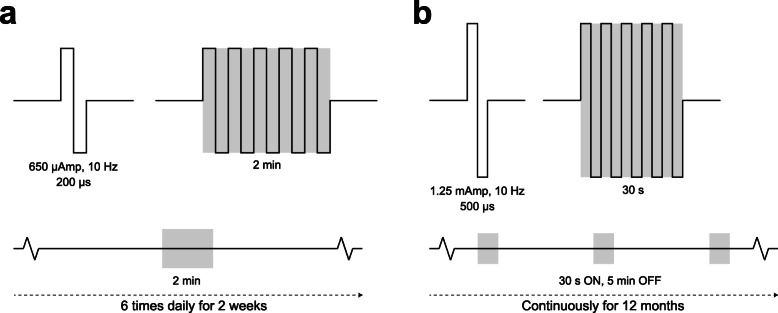
Stimulation parameters. **a**, Stimulation parameters of mouse VNS experiment: 650 μAmp, 10 Hz, pulse width 200 μs for 2 min 6 times daily for 12 days. **b**, Stimulation parameters of human VNS experiment: 1.25 mAmp, 10 Hz, pulse width 500 μs, 30 s ON and 5 min OFF for 12 months. VNS: vagus nerve stimulation

VNS of the left cervical vagus nerve was performed in patients with active Crohn’s disease continuously for 12 months as was described previously (Fig. 1b) (Bonaz et al. 2016; Sinniger et al. 2020). Ileal and colonic biopsies were collected during ileocolonoscopy prior to start of VNS, and 6 and 12 months after start of VNS. These biopsies were used for mRNA expression assessments. In total, 9 patients were included in the study. Of these, 2 patients were removed from the study after a 3-month follow-up because of worsened clinical state. After 1 year of VNS, 5 out of 7 patients were in clinical remission (assessed with the Crohn’s Disease Activity Index, CDAI), and 6 in endoscopic remission (assessed with the Crohn’s Disease Endoscopic Index of Severity, CDEIS).

The study was approved by the Institutional Ethics Review Board (Identifier 11-CHUG-28), registered at ClinicalTrials.gov (NCT01569503), and was conducted in accordance with the Helsinki Declaration and the Good Clinical Practice guidelines of the International Council.

### Animals

Female C57BL/6NCrl inbred mice (10 weeks old) were obtained from Charles River Laboratories, Maastricht, the Netherlands and acclimated for 1 week before performing experiments. The animals were housed under specific pathogen-free conditions in the animal facility at the Amsterdam University Medical Centers, location AMC, Amsterdam, the Netherlands. Animals were maintained on a 12/12 light/dark cycle under constant temperature (20 °C ± 2 °C) and humidity (55%) conditions with ad libitum drinking water and chow. Mice were handled according to the guidelines of the Animal Research Ethics Committee of the University of Amsterdam. Experiments were conducted under a project (application number AVD118002017842; license holder number 11800) approved by the Dutch Central Animal Experiments Committee. Individual experiments were revised and approved by the Animal Research Ethics Committee of the University of Amsterdam. Prior to the experiments protocols were approved by this same committee. A total of 32 mice were used for this study.

### VNS in mice

One hundred micrometer sling cuff electrodes (Micro Cuff Sling, Ref No 1041.2406.51; CorTec GmbH, Freiburg, Germany) were implanted around the left cervical vagus nerve. The electrode was attached to Preci-Dip 1.27 mm 2 Way 1 Row Straight Through (Ref No 702–0092; RS Components B.V., Haarlem, the Netherlands) with PRO Silver Conductive Paint (Ref No 123–9911; RS Components B.V.). The procedure was performed on mice under anesthesia with 2.5% isoflurane in 100% O_2_ (flow 1 L/min). Preoperatively and 24 h postoperatively animals were administered 1 mg/kg meloxicam (Metacam; Boehringer, Ingelheim am Rein, Germany) and 5 mg/kg enrofloxacin (Baytril; Beyer Healthcare, Whippany, NJ, United States of America). Local anesthetics were administered through a lidocaine splash block (2%) before wound closure. Mice recovered for 10 days. At day 0, mice were allocated to the sham group when the impedance measurement of the implanted cuff was above 25 kΩ. Other mice were pair-matched based on weight, after which they were randomly allocated (1:1) to the sham or stim group. The sham group counted 14 mice and the stimulated group counted 16 mice.

Bipolar stimulation was performed for 2 min 6 times daily for 12 days with the following parameters: 650 μAmp, 10 Hz, pulse width 200 μs (Fig. 1a) (Guyot et al. 2019). Proper stimulation was examined by observing behavioral changes of the mice (e.g. altered breathing, decreased movements). Also, before, during, and after the experiment, impedance measurements (using a single frequency of 1 kHz) with a Minirator MR Pro (NTI Audio, Essen, Germany) on the cuff electrodes were performed to ensure proper conduction. Further, the vagus nerves with cuffs from randomly selected mice were collected. After paraffin embedding, a hematoxylin-eosin (HE) staining was conducted to assess damage of the nerve. At the end of the study, mice were euthanized.

### DSS treatment

All animals were treated with dextran sodium sulphate (DSS) to induce acute colitis. 2.25% (w/v) DSS (TdB Consultancy, Uppsala, Sweden) was added to the drinking water for 5 consecutive days. Drinking water with fresh DSS solution was replaced daily. Following DSS-treatment, mice received normal drinking water for 7 days, adding up to a total experiment length of 12 days. During the study, bodyweight and behavior were monitored daily. VNS was started at the same day as DSS treatment.

### mRNA expression analysis

mRNA was extracted from frozen ileal and colonic tissue after lysis in ISOLATE II RNA Lysis Buffer RLY and isolation according to the Bioline ISOLATE II RNA mini kit (both GC biotech b.v. Alphen a/d Rijn, the Netherlands) and cDNA was synthesized by use of the Revertaid first strand cDNA synthesis kit (ThermoFisher Scientific, Landsmeer, the Netherlands). qPCR was performed using SensiFAST SYBR No-ROX (GC biotech b.v.) on a CFX96 Touch™ Real-Time PCR Detection System (Bio-Rad Laboratories B.V., Veenendaal, the Netherlands) and expression levels were analyzed using LinRegPCR software (Ruijter et al. 2009). Expression levels were normalized for reference genes *Eef2*, *Nono*, and *Gapdh* for mouse and *ACTB*, *PSMB6*, and *RPLP0* for human after stability assessment with geNorm (Vandesompele et al. 2002). Primers (all Sigma-Aldrich Chemie N.V., Zwyndrecht, the Netherlands) are listed in Table 1.

**Table 1:** Primer sequences

Gene	Forward sequence (5′ to 3′)	Reverse sequence (5′ to 3′)
*mEef2*	TGTCAGTCATCGCCCATGTG	CATCCTTGCGAGTGTCAGTGA
*mNono*	AAAGCAGGCGAAGTTTTCATTC	ATTTCCGCTAGGGTTCGTGTT
*mGapdh*	ATGTGTCCGTCGTGGATCTGA	ATGCCTGCTTCACCACCTTCT
*mAce2*	TCCAGACTCCGATCATCAAGC	GCTCATGGTGTTCAGAATTGTGT
*mVil1*	CTCAAGACTCCGTCCTGCTG	CCACTTGTTTCTCCGTCCGA
*mCdh1*	AACCCAAGCACGTATCAGGG	GAGTGTTGGGGGCATCATCA
*hACTB*	AATGTGGCCGAGGACTTTGA	TGGCTTTTAGGATGGCAAGG
*hPSMB6*	ACCTGATGGCGGGAATCAT	ATCATACCCCCCATAGGCACT
*hRPLP0*	TGTGGGAGCAGACAATGTGG	TGAGGCAGCAGTTTCTCCAG
*hACE2*	CGAAGCCGAAGACCTGTTCTA	GGGCAAGTGTGGACTGTTCC
*hVIL1*	CCAAAGGCCTGAGTGAAATC	CCTGGAGCAGCTAGTGAACA
*hCDH1*	ATTTTTCCCTCGACACCCGAT	TCCCAGGCGTAGACCAAGA

### Statistical analysis

Graphs were made with GraphPad Prism 8.0 (GraphPad Software, La Jolla, CA, USA) and a Mann-Whitney U test (2 groups) or a Kruskal-Wallis test (> 2 groups) was used to check for statistical significance. In all tests, *P* < 0.05 was accepted as an indication for statistical significance. All data are expressed as median with interquartile range plus the individual data points.

## Results and discussion

### *ACE2* and *TMPRSS2* co-express with nAChRs in stem cells, goblet cells, and enterocytes

**Fig. 2:**
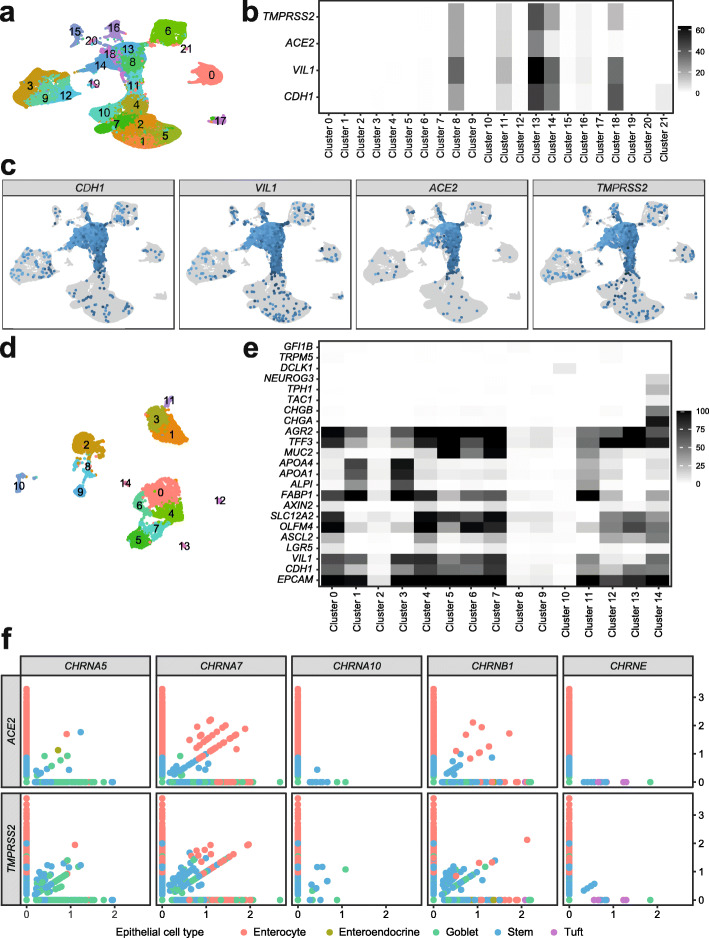
Single-cell RNA-sequencing analysis of ileal epithelial cells. Data were obtained from GSE134809 (Martin et al. 2019). **a** Uniform manifold approximation and projection (UMAP) visualization of the unsupervised clustering analysis of all cells. **b** Heatmap of the percentage cells in a cluster found to be non-zero for epithelial cell markers *VIL1* and *CDH1* as well as SARS-CoV-2-associated genes *ACE2* and *TMPRSS2*. **c** Visualization of the expression of the aforementioned genes on the UMAP. **d** UMAP visualization of the unsupervised clustering analysis of the epithelial cells. **e** Heatmap of the percentage epithelial cells in a cluster found to be non-zero for markers of stem cells (*LGR5*, *ASCL2*, *OLFM4*, *GKN3*, *SLC12A2*, *AXIN2*), goblet cells (*MUC2*, *TFF3*, *CLCA3*, *AGR2*), enterocytes (*FABP1*, *ALPI*, *APOA1*, *APOA4*), enteroendocrine cells (*CHGA*, *CHGB*, *TAC1*, *TPH1*, *NEUROG3*), and tuft cells (*DCLK1*, *TRPM5*, *GFI1B*, *IL25*). **f** Visualization of the normalized counts for the nAChR genes *CHRNA5*, *CHRNA7*, *CHRNA10*, *CHRNB1*, and *CHRNE* on the x-axis against *ACE2* or *TMPRSS2* on the y-axis for all epithelial cells, where each dot represents an individual cell (Martin et al. 2019)

We first investigated which cells express *ACE2* and *TMPRSS2* in the human intestinal context. To this end, we obtained the data from GSE134809 including single-cell RNA-sequencing on inflamed and uninflamed ileal biopsies from patients with Crohn’s disease (Martin et al. 2019). Unsupervised clustering analysis identified 22 clusters (Fig. 2a), with clusters 8, 11, 13, 14, and 18 likely representing the epithelial cells based on their expression of E-cadherin (*CDH1*) and Villin-1 (*VIL1*) (Fig. 2b and c). Expectedly, both *ACE2* and *TMPRSS2* were expressed in the same clusters confirming specific gene expression in epithelial cells in ileum. Subsequent clustering analysis of the epithelial cells yielded 15 subclusters (Fig. 2d), in which we identified stem cells (*LGR5*, *ASCL2*, *OLFM4*, *GKN3*, *SLC12A2*, *AXIN2*), goblet cells (*MUC2*, *TFF3*, *CLCA3*, *AGR2*), enterocytes (*FABP1*, *ALPI*, *APOA1*, *APOA4*), enteroendocrine cells (*CHGA*, *CHGB*, *TAC1*, *TPH1*, *NEUROG3*), and tuft cells (*DCLK1*, *TRPM5*, *GFI1B*, *IL25*) (Fig. 2e) (Haber et al. 2017; Grun et al. 2015). Visualizing the expression of *ACE2* or *TMPRSS2* alongside the expression of the nAChR genes revealed co-expression in stem cells, goblet cells, and enterocytes for *CHRNA5*, *CHRNA7*, *CHRNA10*, *CHRNB1*, and *CHRNE* (encoding nAChR subunits α5, α7, α10, β1, and ε, respectively) (Fig. 2f).

Notably, *ACE2* is clearly expressed in goblet cells, although it has been demonstrated that SARS-CoV and SARS-CoV-2 cannot infect goblet cells in both airway and intestinal epithelia (Lamers et al. 2020). Moreover, the nAChR that was mostly co-expressed with *ACE2* and *TMPRSS2* was α7 nAChR (encoded by *CHRNA7*). This is an essential regulator of inflammation by inhibiting cytokine release through the aforementioned cholinergic anti-inflammatory pathway (Wang et al. 2003). This is relevant because Russo and Leung reported on the role of α7 nAChR in SARS-CoV-2 and demonstrated that smoking results in an upregulation of α7 nAChR leading to an increase of *ACE2 (*Leung et al. 2020a*;* Russo et al. 2020*;* Leung et al. 2020b*)*.

### Chronic electrical VNS does not alter intestinal *ACE2* expression in mice and humans

**Fig. 3:**
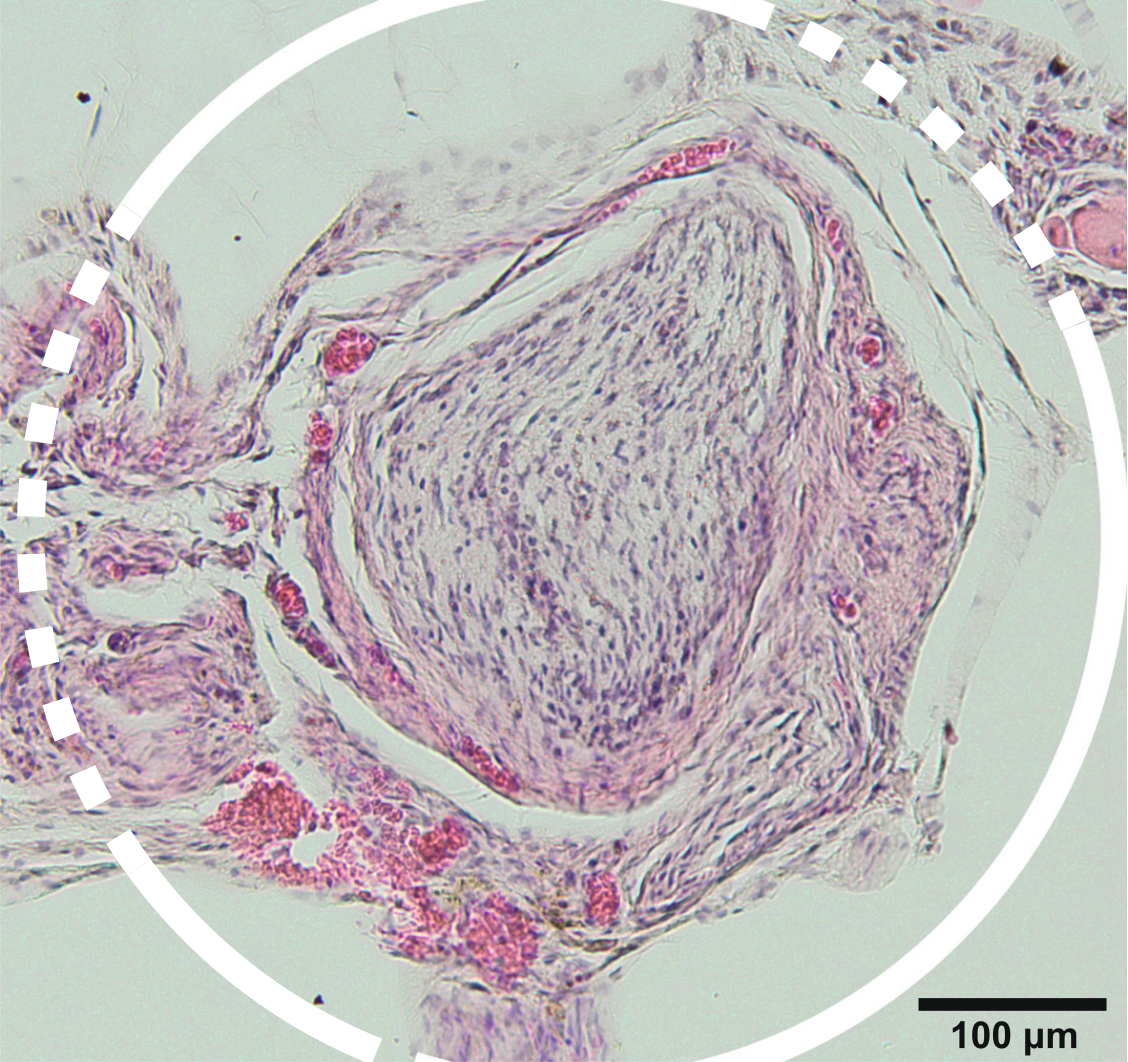
Hematoxylin-eosin (HE) staining of vagus nerve. Image of representative cross-sectioned HE of electrode implanted vagus nerve of VNS-treated mouse (scale bar: 100 μm). White circle indicates the (expected) place of the cuff, that was cleared by sectioning

**Fig. 4:**
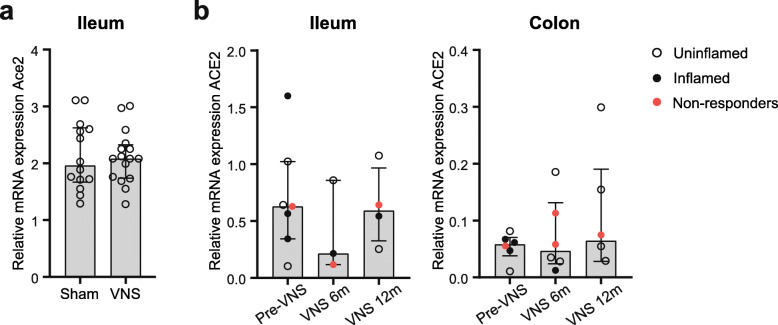
VNS on relative mRNA expression of *ACE2*. **a** Relative mRNA expression of *Ace2* in mouse ileum samples of sham-stimulated (*n* = 14) and stimulated animals (*n* = 16). Levels were corrected for reference genes and epithelial markers E-cadherin (*Cdh1*) and Villin (*Vil1*) to obtain epithelial fraction. **b** Relative mRNA expression of *ACE2* in human ileum (left) and colon (right) samples of patients treated with VNS. Levels were corrected for reference genes and epithelial markers E-cadherin (*CDH1*) and Villin (*VIL1*) to obtain epithelial fraction. mRNA levels of *ACE2* were assessed in biopsies collected prior to VNS, and 6 and 12 months after start VNS**.** Non-responders (red) are patients with no clinical and endoscopic remission. ACE2: Angiotensin-Converting Enzyme 2; VNS: vagus nerve stimulation

As a positive correlation of *ACE2* and *CHRNA7* was previously observed, and chronic cholinergic stimulation typically leads to upregulation of nAChRs, we investigated whether chronic VNS affected the expression of *ACE2 (*Leung et al. 2020b*;* Melroy-Greif et al. 2016*)*. Accordingly, intestinal ileal tissues of mice that were subjected to electrical chronic VNS, applied via an implantable device, were obtained and analyzed for *Ace2* mRNA expression. Adequate VNS was confirmed by behavioral changes of the mice, impedance measurements, and HE stainings not showing any damage of the vagus nerve caused by the cuff electrodes (Fig. 3). Our data show no effect on *Ace2* expression levels. Since samples contained bulk RNA of intestine, values were corrected for epithelial markers *Cdh1* and *Vil1* to determine the epithelial fraction. Also after correction, no differences were observed (Fig. 4a).

Next, we assessed *ACE2* mRNA expression in human intestinal tissues of patients treated with chronic VNS as was described previously (Bonaz et al. 2016; Sinniger et al. 2020). In line with the mouse data, no differential expression was observed between samples collected prior to VNS and 6 and 12 months after start VNS. Correction for *CDH1* and *VIL1*, markers that have shown to be stable under inflammatory conditions (data not shown), did not alter the results (Fig. 4b). Though not significant, a trend towards higher levels of *ACE2* at 12 months VNS could be detected. Noticeably, *ACE2* expression levels in colon samples were relatively low when compared with ileal samples, which is corroborated by earlier literature showing low basal expression levels of *ACE2* in colonic tissue (Qi et al. 2020).

It should be mentioned that in this study we made use of already available tissues. Thus, stimulation parameters such as duration of stimulation, pulse width, and frequency were not optimized for the current research question. In addition, both VNS experiments have been performed in diseased subjects; mice were exposed to DSS inducing colitis, and all humans had active Crohn’s disease. ACE2 has been shown to be upregulated in inflammatory bowel diseases (IBD), and in ileum in particular (Hashimoto et al. 2012; Bangma et al. 2020; Krzysztof et al. 2020). For that reason, in our experiments, basal *ACE2* expression levels might have been higher when compared with healthy subjects because of the colitis background of the tissues, possibly mitigating the results. The considerable divergence between ileal and colonic expression levels substantiates this confounding effect. Though, our analyses did not show significant differences between uninflamed and inflamed samples (Fig. 4b).

Data on the role of α7 nAChRs in colitis are conflicting. Sun et al. demonstrated increased *CHRNA7* expression upon 2,4,6-trinitrobenzenesulfonic acid (TNBS)-induced colitis, whereas Baird et al. found decreased *CHRNA7* expression in IBD (Baird et al. 2016; Sun et al. 2007). Further, in an experimental colitis model, selective α7 nAChR agonists worsened disease (Snoek et al. 2010). These data imply that colitis is associated with *CHRNA7* expression, again modifying the *ACE2* expression levels perchance confusing the current results.

Importantly, when in this manuscript we focus on nicotinic receptors and α7 nAChR specifically, it is important to note that other receptors might play a role in regulating ACE2 as well since VNS unequivocally activates other acetylcholine-binding receptor types. This was not examined in this study.

In addition, we show that VNS does not alter intestinal *ACE2* mRNA expression levels. Protein expression levels, which might be of more importance in this context, have not been assessed. Hence, an effect on SARS-CoV-2 infection could still be observed. This is substantiated by Lamers et al. who demonstrated that SARS-CoV-2 infection of gut enterocytes is independent from the ACE2 expression level (Lamers et al. 2020). Conversely, since SARS-CoV-2 is known to downregulate ACE2 expression, it is plausible that VNS could restore ACE2 levels in infected patients (Glowacka et al. 2010; Kuba et al. 2005). To this end, VNS studies including SARS-CoV-2 infected patients should be performed.

Regardless of the ACE2 expression level, the antagonizing effect of VNS on the devastating systemic cytokine storm might strengthen this intervention as potential treatment for COVID-19. This is greatly underlined by the positive outcomes of the RECOVERY trial studying the effect of low-dose steroid treatment with dexamethasone on SARS-CoV-2 (RECOVERY Collaborative Group et al. 2020). Alike VNS, dexamethasone is acknowledged as inhibitor of the production of pro-inflammatory cytokines (Horton and Remick 2010; Nyhlen et al. 2004).

Concerning the effect of VNS on systemic cytokine responses, a critical note is that these responses depend on the stimulation parameters such as frequency, amplitude, and pulse width as was demonstrated by Tsaava et al., and recently reviewed (Tsaava et al. 2020; Bonaz 2020). Although low frequency stimulation (as was used in our studies) was not examined, stimulation with high amplitudes resulted in an increase in IL-6 and decrease in IL-5 and TNF-α. When refining these parameters in VNS, the subsequent positive regulation of cytokines could substantiate the role of VNS in treating COVID-19.

Finally, granting the potential role for VNS in SARS-CoV-2 infection, one must be aware that parasympathetic neuronal activity through the vagus nerve can induce bronchoconstriction, an absolutely undesirable adverse effect in COVID-19 patients (Belmonte 2005). Even though results on this matter are conflicting, application of VNS in these patients must be performed with great caution (Miner et al. 2012; Steyn et al. 2013). To elucidate and overcome this issue, future studies should focus on more specific stimulation techniques exclusively targeting the cytokine-producing organ of interest.

## Conclusions

The implication of VNS as treatment for SARS-CoV-2 infection has gained high interest. Here, we show that despite gene co-expression of nAChRs, *ACE2*, and *TMPRSS2* in the intestinal epithelium, chronic non-invasive electrical VNS does not significantly alter intestinal *ACE2* expression at a transcriptional level. As bio-electronic neuromodulatory techniques continue to evolve as treatment for controlling inflammation, and with the current SARS-CoV-2 outbreak for COVID-19 in particular, further experimental investigations are needed to shed light on the therapeutic potential of VNS for SARS-CoV-2 infection control.

## Data Availability

Not applicable.

## Abbreviations

ACE2: Angiotensin-Converting Enzyme 2
COVID-19: Coronavirus disease 2019
DSS: Dextran sodium sulphate
HMBG1: High Mobility Group Box 1
IBD: Inflammatory bowel disease
IL: Interleukin
mRNA: Messenger RNA
nAChR: Nicotinic receptor
qPCR: Quantitative polymerase chain reaction
SARS-CoV-2: Severe Acute Respiratory Syndrome Coronavirus 2
TMPRSS2: Transmembrane Serine Protease 2
TNBS: 2,4,6-trinitrobenzenesulfonic acid
TNF: Tumor Necrosis Factor
UMAP: Uniform manifold approximation and projection
VNS: Vagus nerve stimulation

## References

[CR1] Amirian ES (2020). Potential fecal transmission of SARS-CoV-2: current evidence and implications for public health. Int J Infect Dis..

[CR2] Baird A, Coimbra R, Dang X, Eliceiri BP, Costantini TW (2016). Up-regulation of the human-specific CHRFAM7A gene in inflammatory bowel disease. BBA Clin..

[CR3] Bangma A, Voskuil MD, Weersma RK. TNFalpha-antagonist use and mucosal inflammation are associated with increased intestinal expression of SARS-CoV-2 host protease TMPRSS2 in patients with inflammatory bowel disease. Gastroenterology. 2020;S0016-5085(20):34783–1. 10.1053/j.gastro.2020.05.091. 10.1053/j.gastro.2020.05.091 PMC7834454 32553760

[CR4] Belmonte KE (2005). Cholinergic pathways in the lungs and anticholinergic therapy for chronic obstructive pulmonary disease. Proc Am Thorac Soc.

[CR5] Bonaz B (2020). Parameters matter: modulating cytokines using nerve stimulation. Bioelectron Med..

[CR6] Bonaz B, Sinniger V, Hoffmann D, Clarencon D, Mathieu N, Dantzer C (2016). Chronic vagus nerve stimulation in Crohn’s disease: a 6-month follow-up pilot study. Neurogastroenterol Motil..

[CR7] Butler A, Hoffman P, Smibert P, Papalexi E, Satija R (2018). Integrating single-cell transcriptomic data across different conditions, technologies, and species. Nat Biotechnol..

[CR8] Creamer MR, Wang TW, Babb S, Cullen KA, Day H, Willis G (2019). Tobacco product use and cessation indicators among adults - United States, 2018. MMWR Morb Mortal Wkly Rep..

[CR9] Farsalinos K, Niaura R, Le Houezec J, Barbouni A, Tsatsakis A, Kouretas D (2020). Editorial: Nicotine and SARS-CoV-2: COVID-19 may be a disease of the nicotinic cholinergic system. Toxicol Rep.

[CR10] Fudim M, Qadri YJ, Ghadimi K, MacLeod DB, Molinger J, Piccini JP, et al. Implications for neuromodulation therapy to control inflammation and related organ dysfunction in COVID-19. J Cardiovasc Transl Res. 2020:1–6. 10.1007/s12265-020-10031-6. 10.1007/s12265-020-10031-6 PMC7250255 32458400

[CR11] Glowacka I, Bertram S, Herzog P, Pfefferle S, Steffen I, Muench MO (2010). Differential downregulation of ACE2 by the spike proteins of severe acute respiratory syndrome coronavirus and human coronavirus NL63. J Virol..

[CR12] Grun D, Lyubimova A, Kester L, Wiebrands K, Basak O, Sasaki N (2015). Single-cell messenger RNA sequencing reveals rare intestinal cell types. Nature..

[CR13] Guyot M, Simon T, Panzolini C, Ceppo F, Daoudlarian D, Murris E (2019). Apical splenic nerve electrical stimulation discloses an anti-inflammatory pathway relying on adrenergic and nicotinic receptors in myeloid cells. Brain Behav Immun..

[CR14] Haber AL, Biton M, Rogel N, Herbst RH, Shekhar K, Smillie C (2017). A single-cell survey of the small intestinal epithelium. Nature..

[CR15] Hamming I, Timens W, Bulthuis ML, Lely AT, Navis G, van Goor H (2004). Tissue distribution of ACE2 protein, the functional receptor for SARS coronavirus. A first step in understanding SARS pathogenesis. J Pathol..

[CR16] Hashimoto T, Perlot T, Rehman A, Trichereau J, Ishiguro H, Paolino M (2012). ACE2 links amino acid malnutrition to microbial ecology and intestinal inflammation. Nature..

[CR17] Hoffmann M, Kleine-Weber H, Schroeder S, Kruger N, Herrler T, Erichsen S (2020). SARS-CoV-2 cell entry depends on ACE2 and TMPRSS2 and is blocked by a clinically proven protease inhibitor. Cell.

[CR18] Horton DL, Remick DG (2010). Delayed addition of glucocorticoids selectively suppresses cytokine production in stimulated human whole blood. Clin Vaccine Immunol..

[CR19] Kuba K, Imai Y, Rao S, Gao H, Guo F, Guan B (2005). A crucial role of angiotensin converting enzyme 2 (ACE2) in SARS coronavirus-induced lung injury. Nat Med..

[CR20] Lamers MM, Beumer J, van der Vaart J (2020). SARS-CoV-2 productively infects human gut enterocytes. Science..

[CR21] Leung JM, Yang CX, Sin DD (2020). COVID-19 and nicotine as a mediator of ACE-2. Eur Respir J.

[CR22] Leung JM, Yang CX, Tam A, Shaipanich T, Hackett TL, Singhera GK (2020). ACE-2 expression in the small airway epithelia of smokers and COPD patients: implications for COVID-19. Eur Respir J..

[CR23] Luecken MD, Theis FJ (2019). Current best practices in single-cell RNA-seq analysis: a tutorial. Mol Syst Biol..

[CR24] Martin JC, Chang C, Boschetti G, Ungaro R, Giri M, Grout JA (2019). Single-cell analysis of Crohn’s disease lesions identifies a pathogenic cellular module associated with resistance to anti-TNF therapy. Cell.

[CR25] Melroy-Greif WE, Stitzel JA, Ehringer MA (2016). Nicotinic acetylcholine receptors: upregulation, age-related effects and associations with drug use. Genes Brain Behav..

[CR26] Miner JR, Lewis LM, Mosnaim GS, Varon J, Theodoro D, Hoffmann TJ (2012). Feasibility of percutaneous vagus nerve stimulation for the treatment of acute asthma exacerbations. Acad Emerg Med..

[CR27] Nestorowa S, Hamey FK, Pijuan Sala B, Diamanti E, Shepherd M, Laurenti E (2016). A single-cell resolution map of mouse hematopoietic stem and progenitor cell differentiation. Blood..

[CR28] Nowak JK, Lindstrøm JC, Kalla R, Ricanek P, Halfvarson J, Satsangi J (2020). Age inflammation and disease location are critical determinants of intestinal expression of SARS-CoV-2 receptor ACE2 and TMPRSS2 in inflammatory bowel disease. Gastroenterology.

[CR29] Nyhlen K, Gautam C, Andersson R, Srinivas U (2004). Modulation of cytokine-induced production of IL-8 in vitro by interferons and glucocorticosteroids. Inflammation..

[CR30] Petrilli CM, Jones SA, Yang J, Rajagopalan H, O’Donnell L, Chernyak Y (2020). Factors associated with hospital admission and critical illness among 5279 people with coronavirus disease 2019 in New York City: prospective cohort study. BMJ..

[CR31] Qi F, Qian S, Zhang S, Zhang Z (2020). Single cell RNA sequencing of 13 human tissues identify cell types and receptors of human coronaviruses. Biochem Biophys Res Commun..

[CR32] R Core Team (2016). R: A language and environment for statistical computing.

[CR33] RECOVERY Collaborative Group, Horby P, Lim WS, et al. Dexamethasone in Hospitalized Patients with Covid-19 - Preliminary Report. N Engl J Med. 2020:NEJMoa2021436. 10.1056/NEJMoa2021436.

[CR34] Richardson CE, Morgan JM, Jasani B, Green JT, Rhodes J, Williams GT (2003). Effect of smoking and transdermal nicotine on colonic nicotinic acetylcholine receptors in ulcerative colitis. QJM..

[CR35] Ruijter JM, Ramakers C, Hoogaars WM, Karlen Y, Bakker O, van den Hoff MJ (2009). Amplification efficiency: linking baseline and bias in the analysis of quantitative PCR data. Nucleic Acids Res..

[CR36] Russo P, Bonassi S, Giacconi R, Malavolta M, Tomino C, Maggi F (2020). COVID-19 and smoking. Is nicotine the hidden link?. Eur Respir J.

[CR37] Sinniger V, Pellissier S, Fauvelle F, Trocme C, Hoffmann D, Vercueil L, et al. A 12-month pilot study outcomes of vagus nerve stimulation in Crohn’s disease. Neurogastroenterol Motil. 2020:e13911. 10.1111/nmo.13911. 10.1111/nmo.13911 32515156

[CR38] Snoek SA, Verstege MI, van der Zanden EP, Deeks N, Bulmer DC, Skynner M (2010). Selective alpha7 nicotinic acetylcholine receptor agonists worsen disease in experimental colitis. Br J Pharmacol..

[CR39] Staats P, Giannakopoulos G, Blake J, Liebler E, Md R (2020). Use of non-invasive vagus nerve stimulation to treat respiratory symptoms associated with COVID-19: a theoretical hypothesis and early clinical experience. Neuromodulation..

[CR40] Steyn E, Mohamed Z, Husselman C (2013). Non-invasive vagus nerve stimulation for the treatment of acute asthma exacerbations-results from an initial case series. Int J Emerg Med..

[CR41] Stuart T, Butler A, Hoffman P, Hafemeister C, Papalexi E, Mauck WM (2019). Comprehensive integration of single-cell data. Cell.

[CR42] Summers AE, Whelan CJ, Parsons ME (2003). Nicotinic acetylcholine receptor subunits and receptor activity in the epithelial cell line HT29. Life Sci..

[CR43] Sun YP, Wang HH, He Q, Cho CH (2007). Effect of passive cigarette smoking on colonic alpha7-nicotinic acetylcholine receptors in TNBS-induced colitis in rats. Digestion..

[CR44] Team CC-R (2020). Preliminary estimates of the prevalence of selected underlying health conditions among patients with coronavirus disease 2019 - United States, February 12-March 28, 2020. MMWR Morb Mortal Wkly Rep..

[CR45] Tracey KJ (2002). The inflammatory reflex. Nature..

[CR46] Tsaava T, Datta-Chaudhuri T, Addorisio ME, Masi EB, Silverman HA, Newman JE (2020). Specific vagus nerve stimulation parameters alter serum cytokine levels in the absence of inflammation. Bioelectron Med..

[CR47] Vandesompele J, De Preter K, Pattyn F, Poppe B, Van Roy N, De Paepe A (2002). Accurate normalization of real-time quantitative RT-PCR data by geometric averaging of multiple internal control genes. Genome Biol.

[CR48] Wang H, Yu M, Ochani M, Amella CA, Tanovic M, Susarla S (2003). Nicotinic acetylcholine receptor alpha7 subunit is an essential regulator of inflammation. Nature..

[CR49] Wickham H (2016). ggplot2: elegant graphics for data analysis.

[CR50] Xiao F, Tang M, Zheng X, Liu Y, Li X, Shan H (2020). Evidence for gastrointestinal infection of SARS-CoV-2. Gastroenterology..

[CR51] Yang L, Tu L. Implications of gastrointestinal manifestations of COVID-19. Lancet Gastroenterol Hepatol. 2020; 10.1016/S2468-1253(20)30132-1, https://www.thelancet.com/journals/langas/article/PIIS2468-1253(20)30132-1/fulltext. 10.1016/S2468-1253(20)30132-1 PMC7217632 32405602

[CR52] Zhang B, Zhou X, Qiu Y, Feng F, Feng J, Jia Y, et al. Clinical characteristics of 82 death cases with COVID-19. medRxiv. 2020; 10.1101/2020.02.26.20028191.

